# Preferential killing of melanoma cells by a p16-related peptide

**DOI:** 10.1242/bio.059965

**Published:** 2023-08-17

**Authors:** Julia K. Soo, Joanna T. Castle, Dorothy C. Bennett

**Affiliations:** Molecular & Clinical Sciences Research Institute, St George's, University of London, Cranmer Terrace, London SW17 0RE, UK

**Keywords:** Cell-penetrating peptide, Melanoma, Therapy, p16 (CDKN2A), Apoptosis, Senescence

## Abstract

We report the identification of a synthetic, cell-penetrating peptide able to kill human melanoma cells efficiently and selectively, while being less toxic to normal human melanocytes and nontoxic to human fibroblasts. The peptide is based on the target-binding site of the melanoma suppressor and senescence effector p16 (also known as INK4A or CDKN2A), coupled to a cell-penetrating moiety. The killing is by apoptosis and appears to act by a route other than the canonical downstream target of p16 and CDK4, the retinoblastoma (RB) protein family, as it is also effective in HeLa cells and a melanocyte line expressing HPV E7 oncogenes, which both lack any active RB. There was varying toxicity to other types of cancer cell lines, such as glioblastoma. Melanoma cell killing by a p16-derived peptide was reported once before but only at a higher concentration, while selectivity and generality were not previously tested.

## Teaser

**Summary:** The prospect for metastatic melanoma patients remains poor despite novel therapies; co-therapies are needed. We describe a peptide that efficiently kills metastatic melanoma cell lines yet spares normal fibroblasts.

## INTRODUCTION

The protein p16, also called inhibitor of kinase 4, A (INK4A) or cyclin-dependent kinase inhibitor 2A (CDKN2A), is encoded by *CDKN2A*, the commonest known susceptibility gene for melanoma (Landi et al., 2020; Bennett, 2016; Castaneda-Garcia et al., 2022). *CDKN2A* is also one of the two genes reported to be most commonly defective or deleted in human cancers generally, the other being *TP53*, encoding p53 (Ben-Porath and Weinberg, 2005). Both p16 and p53 are major intermediates in cell senescence, a powerful tumour suppressor mechanism in the form of a permanent proliferative arrest that occurs after extensive normal cell proliferation and telomere shortening, or after oncogene activation or other stresses (Chandler and Peters, 2013; Gorgoulis et al., 2019; Wang et al., 2022). p16 function is commonly impaired as a relatively early step in the progression of cancers, including melanoma, attributed to the need for cells to evade senescence for a sizeable lesion to form (Bennett, 2016; Iacobuzio-Donahue et al., 2012; Shain et al., 2018; Witkiewicz et al., 2011). p16 has a particular (although incompletely understood) link to melanoma, as *CDKN2A* is a susceptibility gene almost specifically for melanoma, while p16 is also deleted, mutated or silenced in around 80% of uncultured sporadic invasive melanomas (Shain et al., 2018; Bennett, 2016). *CDKN2A* also encodes another growth suppressor, ARF, but ARF function is less commonly impaired by familial melanoma mutations in the gene than that of p16. In comparison, p53 is both wild type in sequence and expressed in most uncultured growing melanomas (Bennett, 2016; Hodis et al., 2012; MacKenzie Ross et al., 2013; Shain et al., 2018).

The established, canonical molecular action of p16 is inhibition of cyclin-dependent kinase (CDK) 4 and CDK6. CDK4 and CDK6 thereby fail to phosphorylate and inactivate the retinoblastoma (RB) protein family (RB1/pRB, RBL1/p107 and RBL2/p130, hereafter jointly designated as RB), resulting in sustained RB activation and cell-cycle arrest (Chandler and Peters, 2013). There is little or no p16 expression in normal, young tissues and p16-mediated arrest appears to be largely specific to cell senescence, being associated with tumour suppression and also ageing (Chandler and Peters, 2013; Witkiewicz et al., 2011; Gorgoulis et al., 2019). CDK4 can also phosphorylate and activate a second major substrate, the master G2/M transcription factor FOXM1 (Anders et al., 2011), which is thus another potential target of inhibition by p16. FOXM1 can suppress cell senescence upon overexpression (Anders et al., 2011). The specific importance of cell senescence in melanoma is further highlighted by the realization that many familial melanoma susceptibility genes have a connection with cell senescence (Castaneda-Garcia et al., 2022). One of these genes also harbours the commonest known type of mutation in advanced sporadic melanoma (85% in metastatic lesions), namely, activating promoter mutations of the telomerase catalytic subunit gene *TERT*, required for cell immortality (Horn et al., 2013; Huang et al., 2013; Shain et al., 2018).

Cell senescence reinduction presents intriguing possibilities as a novel modality in cancer therapy (Cairney et al., 2012; Gorgoulis et al., 2019; Wang et al., 2022). As the great majority of melanoma cells have lost p16 function, restoration of p16 to them appears to be a promising avenue for such therapeutic cell senescence, one which we therefore decided to explore. Gene transfer is not an auspicious route for clinical cancer therapy, as 100% transfer efficiency is unlikely and unaltered cancer cells could grow back. However, another highly specific approach, potentially more adaptable to the clinic, yet probably under-explored, is the use of ‘designer’ peptides: small peptides that mimic part of a desired protein, and which can have highly specific effects. These can include terminal cell-penetrating peptides or carrier sequences, often arginine-rich, that enable permeation through cell membranes (Madani et al., 2011).

We are aware of three previous studies on the effects of cell-penetrating p16-mimetic peptides on cells. Fåhraeus and colleagues (1996) reported that short peptides from the CDK4-binding site of human p16 could bind and inhibit CDK4 *in vitro*, with highest activity from their 20-amino-acid ‘peptide 6’. Binding was further strengthened by a point substitution in this peptide, D92A (residue 92 as numbered in the complete p16 sequence). Moreover, when penetratin, a cell-penetrating peptide from the *Drosophila* Antennapedia protein, was covalently added, the p16 peptide could enter cultured human HaCaT keratinocytes and rapidly inhibit entry into S phase (Fåhraeus et al., 1996). This was confirmed by Gius et al. (1999), also using HaCaT cells and the Fåhraeus peptide 6, now linked to the HIV TAT protein for cell penetration. Cells were treated for up to 15 h (in a study of the requirement for CDK4 for exit from G1 phase). Another group (Noonan et al., 2005) likewise reported that two other peptides containing p16-derived and penetratin-derived sequences, used at 50 µM, could inhibit the net growth of two melanoma cell lines. More surprisingly, the peptides induced apoptosis in these cells at 24-48 h, not just arrest. They reported reduced tumour formation by one of the two melanoma lines upon xenografting to immunodeficient mice with injections of one such p16 peptide. This interesting work appears not to have been pursued further, however. The effects on normal cells versus immortal or malignant cells have not been compared, nor the mechanism of action of the peptides elucidated.

We now report the efficient apoptotic killing of 3/3 lines of human metastatic melanoma cells by a 28-mer p16-based cell-penetrating peptide, at 30 µM or less, over 5 days. The peptide also kills HeLa cervical cancer cells and melanocytes expressing the HPV-16 E7 oncogene, surprisingly indicating that this killing effect does not require activity of CDK4 or the RB family. It is less cytotoxic to melanocytes than to melanoma cells, and variably toxic to other cancer cell lines. Most remarkably, this peptide has no detectable effect on the growth of normal human fibroblasts, at a concentration lethal to melanoma cells and HeLa cells.

## RESULTS

### Killing of melanoma cells by active peptide P16P1

The three p16-related peptides studied are shown in the Materials and Methods. P16P1, the test peptide, contains peptide 6 from Fåhraeus et al. (1996) with their D92A substitution to increase target binding. Here, we added an eight-arginine (R) tail (giving 9×R with the natural R at residue 103 of p16) for cell penetration. P16P2 is a control peptide without the D92A substitution and with an L97R substitution found in a melanoma-susceptible family and reported to impair the binding of p16 to CDK4 (Soufir et al., 1998). P16P3 is another control peptide, identical to P16P1 except without the 8R tail, thus not expected to enter cells.

**Fig. 1.**
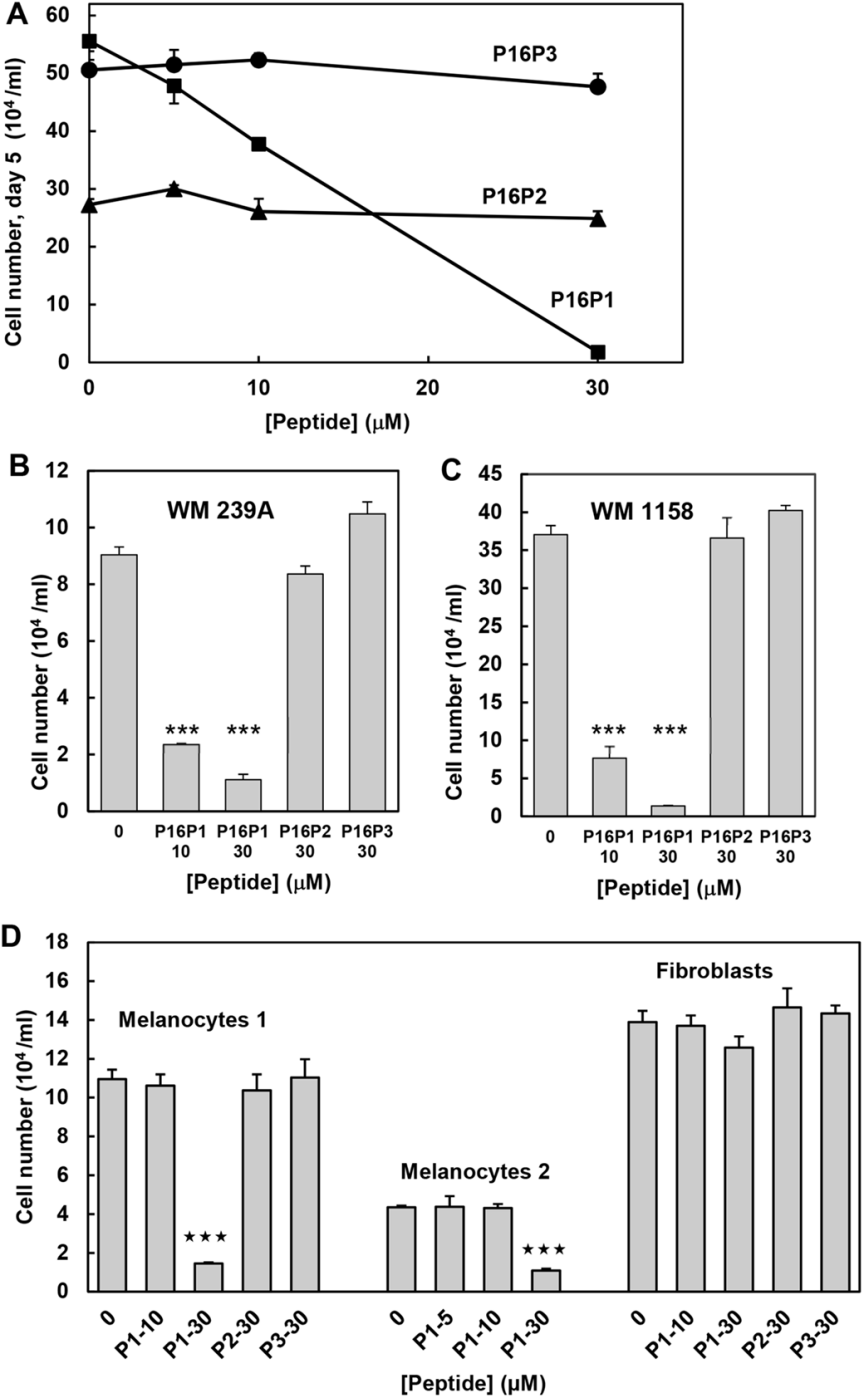
**Representative cell number responses to active (P16P1) and control peptides (P16P2 and P16P3).** Melanoma cells were plated at 2×10^4^ ml^−1^, normal melanocytes at 5×10^4^ ml^−1^ and fibroblasts at 3×10^4^ ml^−1^ (such that control cells did not reach saturation density in the set time). Triplicate haemocytometer counts from each of triplicate cultures were taken after 5 days, or 7 days for normal melanocytes. Mean and s.e.m. of culture means are shown. ‘0’ indicates vehicle control. (A) 451Lu metastatic melanoma cells, dose-response plots for all three peptides. (B,C) WM239A (B) and WM1158 (C) metastatic melanoma cells. (D) Normal human cells, counted at day 7 (melanocytes) or day 5 (fibroblasts). Peptide names are abbreviated. Melanocytes 1 and 2 were strains Nohm-1 and 830c. The fibroblasts were dermal strain Hfib. Differences from vehicle control were tested by one-tailed, unpaired Student's *t*-tests. ***P<0.001.

**Fig. 2.**
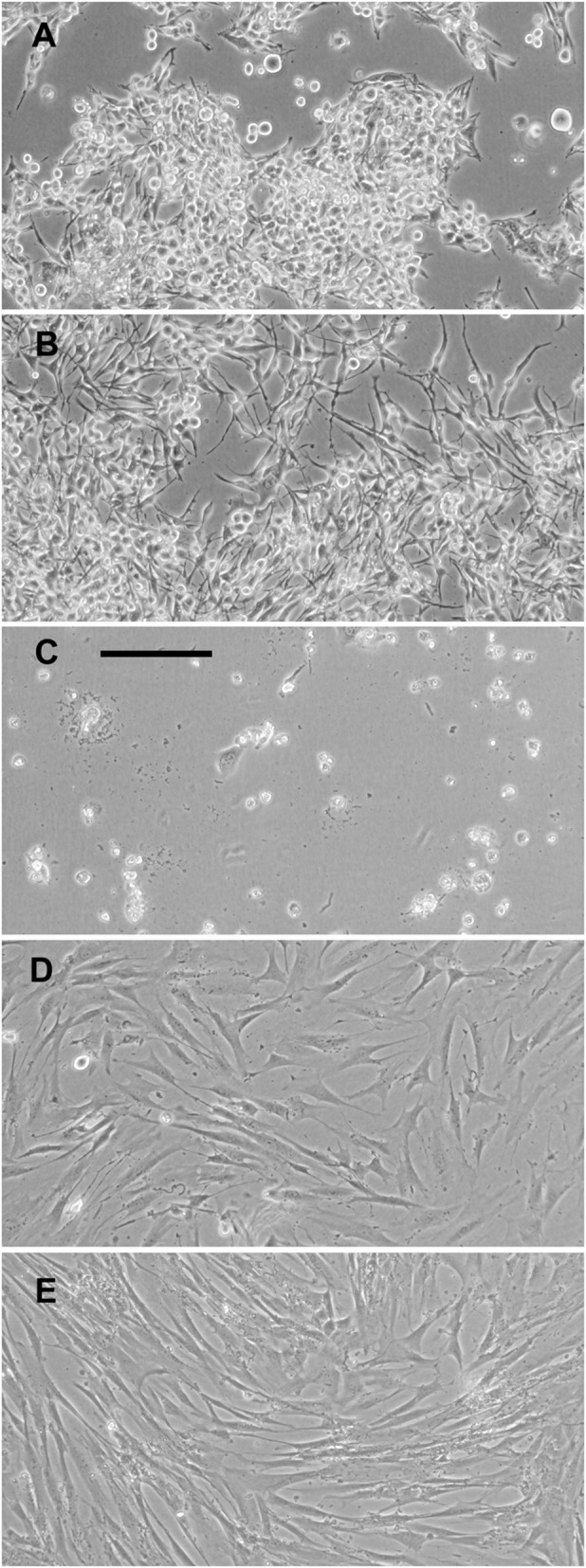
**Morphological effects of P16P1 observed for melanoma cells but not fibroblasts.** Representative phase-contrast images of cells after 5 days of growth with or without P16P1. (A-C) 451Lu melanoma cells with vehicle control (A), or P16P1 at 10 µM (B) and 30 µM (C). In C, a few cells remain but appear dead. (D,E) Hfib dermal fibroblasts with vehicle control (D) or 30 µM P16P1 (E), showing no apparent effect of the peptide. Images are representative of three or more independent experiments. Scale bar: 100 µm for all panels.

Melanoma adjuvant therapies target metastatic cells, so initial studies were done with the highly metastatic human melanoma line 451Lu, selected for metastasis by passage through mice (Herlyn et al., 1990). As Figs 1A and 2A,C show, culture of 451Lu cells for 5 days with peptide P16P1 resulted in a dramatic, dose-dependent reduction of cell numbers. Cell killing by 30 µM P16P1 was immediately suggested by the appearance of the cells and the reduction of cell numbers far below the plating density. Neither control peptide had any significant effect over the same concentration range, indicating that cell penetration and wild-type Leu97 in the CDK4-binding site were needed for the toxic effect. Similar effects of the three peptides were observed with two other metastatic melanoma lines, WM239A and WM1158 (Fig. 1B,C). All three melanoma lines had genetic defects in p16. The status of p16 pathway components in these and all other lines used in this study is shown in Table S1 for reference. 451Lu cells grown with the intermediate concentration of 10 µM P16P1 appeared somewhat larger and flatter than control cells (Fig. 2A,B), and a few of them expressed high levels of β-galactosidase, a lysosomal enzyme abundant in senescent cells (Gorgoulis et al., 2019) (data not shown). Accordingly, there may have been induction of senescence by 10 µM P16P1 in a small minority of cells. However, this was unremarkable compared to the extensive cell death with 30 µM peptide, and was therefore not investigated further.

### Reduced effects of P16P1 peptide on normal cells and lines from other cancer types

To determine whether the killing effect had any specificity for malignant cells, we tested the three peptides on normal human dermal fibroblasts and two strains of normal human melanocytes. Normal fibroblasts, remarkably, showed no detectable response at all to the peptide, even at 30 µM, with which most melanoma cells were dead by day 5. Cell morphology appeared normal (Fig. 2D,E). Intriguingly, there was some toxicity of P16P1 to both strains of normal melanocytes (Fig. 1D), with substantially reduced numbers at the higher concentration of 30 µM, although there was no significant effect on either strain at 10 µM (which did deplete melanoma cells). We then tested some other human cancer cell types. The P16P1 peptide showed varying abilities to kill or inhibit these, with cell number reductions at 30 µM of about 80% in a glioblastoma cell line, 55% in a colon cancer line and 27% in a prostate cancer line (Fig. S1). The data suggest additional toxicity for pigment cells, whether normal or malignant, as well as higher toxicity for cancer versus normal cells.

### Apoptotic nature of the cell death

**Fig. 3.**
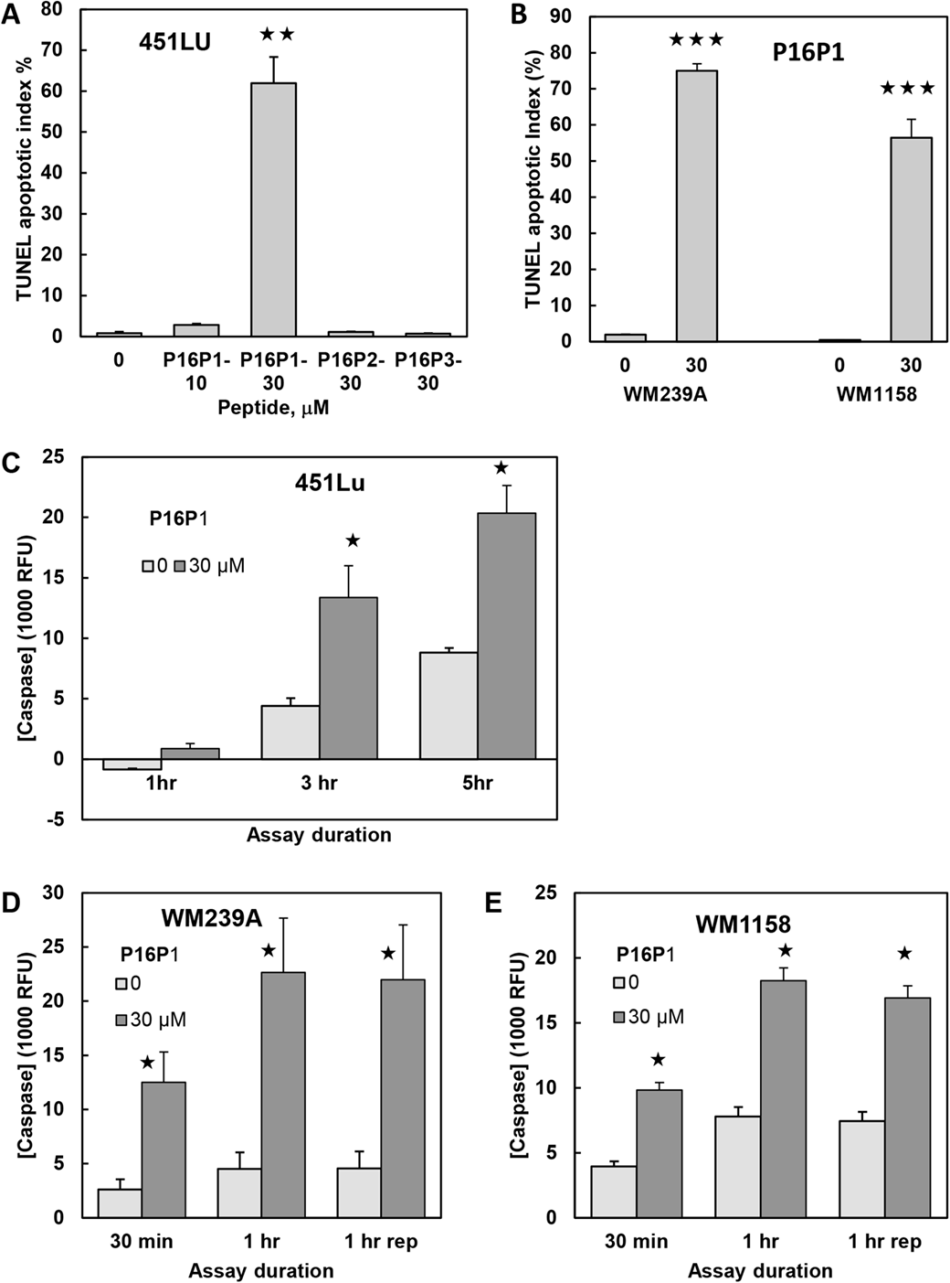
**Apoptosis induced by P16P1 in melanoma cells.** Assays were performed after 2 days, as there were insufficient cells left after 5 days. Cell lines, peptides and concentrations are indicated. For TUNEL assays, three microscope fields were counted and averaged from each of triplicate cultures per treatment. Charts show mean and s.e.m. of culture means. (A) Representative TUNEL assays of 451Lu cells showing very high positivity with P16P1 (30 µM) only. (B) Representative TUNEL assays of WM239A and WM1158 melanoma cells, likewise showing high positivity with P16P1. (C-E) Caspase 3/7 assays showing significant increases with P16P1 in 451Lu cells (C), WM239A cells (D) and WM1158 cells (E). RFU, relative fluorescence units. Mean and s.e.m. of triplicate cultures are shown. Assay duration indicates the time of incubation in the actual assay; ‘rep’ indicates an independently repeated experiment. Differences from vehicle controls were tested by one-tailed, unpaired Student's *t*-tests. *P<0.05; **P<0.01; ***P<0.001.

We then investigated whether the observed cell death was attributable to apoptosis. Two different assays of apoptosis were used, terminal deoxynucleotidyl transferase dUTP nick-end labelling (TUNEL) and caspase 3/7 assays. These were performed after 2 days of culture with peptides (when enough melanoma cells were still alive). Both apoptotic markers showed large and significant increases in all three melanoma lines after culture with P16P1 (Fig. 3). No effects of the control peptides P16P2 and P16P3 on apoptosis were detectable (Fig. 3A), which was expected as they did not affect cell number.

### Lack of requirement for RB-family proteins

**Fig. 4.**
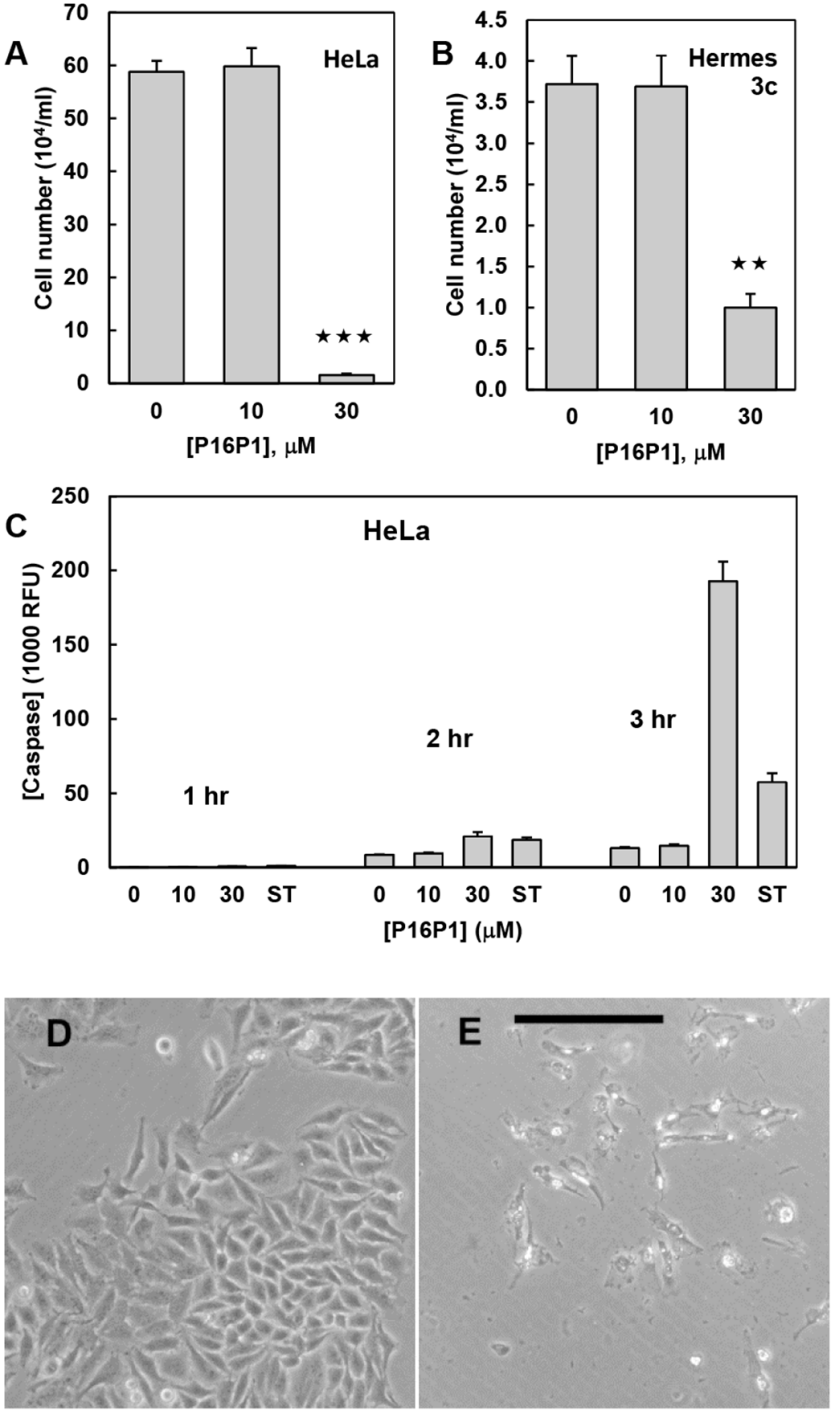
**Killing of HeLa cells and HPV-E7-expressing melanocytes by P16P1.** (A,B) Reductions of cell number below the plating density for HeLa cells (A) and Hermes 3c immortal human melanocytes (B). HeLa cells were plated at 2×10^4^ cells ml^−1^ and counted after 5 days, whereas Hermes 3c were plated at 3×10^4^ cells ml^−1^ and counted after 5 days; media were renewed at 3 days. Mean and s.e.m. of culture means are shown. **P<0.01; ***P<0.001. (C) Caspase 3/7 assays of HeLa cells performed after 2 days of culture and showing substantial apoptosis with 30 µM P16P1, more than with the positive control of 0.5 µM staurosporine (ST). Mean and s.e.m. of triplicate cultures are shown. (D,E) Morphology of HeLa cells after 5 days with vehicle control (D) or 30 µM P16P1 (E). A few cells in E are still attached but they appear dead. Images are representative of at least three independent experiments. Scale bar: 200 µm.

To investigate further whether the toxicity was through CDK4 and the RB family, we tested P16P1 on HeLa cells and Hermes 3c melanocytes. HeLa cervical carcinoma cells carry human papilloma virus 18 (HPV18) and thus express its oncogenes E6 and E7 (DeFilippis et al., 2003). E7 blocks both the best-known cell-arrest route of p16 through the RB family and the above-mentioned potential route through lack of FOXM1 activation, as E7 mimics CDK4 both by inactivating the whole RB family (DeFilippis et al., 2003) and by activating FOXM1 (formerly called MPP2) (Lüscher-Firzlaff et al., 1999). HeLa cells express high levels of normal p16, so that CDK4 is expected to be completely inhibited already in these cells. Accordingly, if P16P1 toxicity works through CDK4 and the RB family, the effects should be absent in HeLa cells. Fig. 4A,C-E shows, however, the efficient killing of HeLa cells by P16P1 at 30 µM. Likewise the human melanocyte line Hermes 3c, an immortal Nohm-1 subline expressing the similar E7 oncogene of HPV16 (Gray-Schopfer et al., 2006), was also largely killed at 30 µM (Fig. 4B), to a similar extent to the non-immortal melanocyte lines (Fig. 1D). These data strongly suggest that the cytotoxic effect of peptide P16P1 is not mediated by the RB family, unlike the senescence effect of normal, full-length p16.

## DISCUSSION

The finding of Noonan et al. (2005) that a p16-derived peptide can kill rather than arrest melanoma cells was interesting and unexpected, based on the normal action of p16 of cell-cycle arrest and senescence, and has not been subsequently validated to our knowledge. Here, we show that a different p16-derived peptide, P16P1, can also efficiently kill melanoma cells (3/3 lines) and report for the first time that the toxicity is greatest for melanoma cells compared to various other cancer cells and normal melanocytes. Moreover, this peptide has no detectable effect at all on normal fibroblasts, whereas it does kill melanoma cells, HeLa cervical cancer cells and a glioblastoma line (less so a prostate carcinoma and a colon carcinoma line). The peptide also appears more toxic for melanocytes than fibroblasts. These intriguing specificities clearly merit further investigation.

A possibility for the lack of action of P16P1 on human dermal fibroblasts could be that it failed to enter this one cell line, out of the nine tested. However, we showed previously, via conjugation to the fluorophore 5-carboxytetramethylrhodamine (TAMRA), that octoarginine enabled efficient entry of similarly sized peptides into human dermal fibroblasts as well as melanocytes (Castle, 2018). Polyarginine-coupled and arginine-rich peptides have likewise been found by other authors and ourselves to enter all tested mammalian cell types out of a wide range, including various mouse, human and simian fibroblasts and other mesodermal cells, as well as normal and malignant epithelial and blood cell lineages (e.g. Mi et al., 2000; Kim et al., 2009; Izumisawa et al., 2011). Lack of entry of P16P1 into dermal fibroblasts thus seems unlikely. Future tests of this and related peptides on additional normal cell types will be of much interest, regarding toxicity as well as physical effects such as entry.

The cell killing by P16P1 required entry into cells, was prevented by a mutation in the CDK4-binding site and was apoptotic in nature. We report, however, that the cytotoxic effect was apparently not mediated by the RB family, the canonical targets of CDK4, as the peptide also efficiently killed two lines of cells expressing HPV E7 oncogenes, which deplete cells of all three of the RB family. This is surprising, as the peptide is from the CDK4-binding site of p16 and no cells were killed by peptide P16P2 with the L97R mutation, reported to abrogate CDK4 binding. The killing is unlikely to result from the D92A substitution in P16P1 that increases its CDK4-inhibitory activity (Fåhraeus et al., 1996), as the p16 peptides of Noonan et al. (2005) also killed melanoma cells, yet lacked that substitution. Noonan et al. (2005) did observe reduced killing of an RB1-null melanoma line by their p16-related peptide at 50 µM, and we saw a lesser sensitivity of HeLa cells to P16P1 compared to melanoma cells, at 10 µM but not at 30 µM. However, it remains unlikely that CDK4 inhibition mediates the death because, as mentioned, CDK4 is already expected to be completely inhibited by normal p16 in HeLa cells and, moreover, CDK4 inhibition normally arrests rather than kills cells. It seems more likely that P16P1 acts through a second molecular target. Other molecular actions of full-length normal p16 have been reported, including inhibition of CDK7 (Nishiwaki et al., 2000) and of NFκB-RELA (Becker et al., 2005). Either action could plausibly kill cancer cells, as RELA upregulates at least three cell survival pathways in melanoma cells (reviewed by Bennett, 2008) and CDK7 is a vital cell-cycle kinase. Interestingly, a subset of p16 mutations from melanoma families could disrupt the inhibition of RELA as well as that of CDK4, suggesting that this normal p16 action may also have some negative effect on melanoma (Becker et al., 2005). Perhaps the L97R mutation, as in P16P2, also affects RELA binding and/or CDK7 binding. Alternatively, there may be another unknown target(s) of p16 or a new target of the peptide not shared with normal p16. Further investigation of these points in a new project will be important, although we wish to share the interesting findings at this point.

Noonan et al. (2005) reported that a different cell-penetrating p16 peptide could reduce growth of xenografted human melanoma in mice. This gives support for clinical applicability of this type of agent, with apparent sparing of normal tissues at relevant dose levels (Noonan et al., 2005). The main molecular target of the cancer-cytotoxic action of P16P1 may require extensive study to pin down. Nonetheless, the current findings already seem very interesting: a novel agent that can efficiently kill melanoma cells at a concentration that completely spares some normal cells. Future development should involve a search for a variant or concentration that preserves the antimelanoma action while more effectively sparing normal melanocytes, ideally through understanding the specificity for pigment cells, and testing *in vivo* to assess effects on other normal cell types.

## MATERIALS AND METHODS

### Cell culture

Melanoma lines were originally obtained from Meenhard Herlyn (Wistar Institute, PA, USA), and 830c human melanocytes from Zalfa Abdel-Malek (University of Cincinnati, OH, USA). Other cell strains were derived by us or obtained as listed in Fig. S1. All lines were validated and checked for contamination, recent to the time of experimentation. All three melanoma lines were deleted or mutant for *CDKN2A* and wild type for *CDK4.* The status of p16 pathway components in the cell lines used in this study is shown for reference in Table S1.

All cells were grown at 37°C in humidified incubators gassed with 10% CO_2_ in air, and media were changed every 3-4 days. All lines were pleuropneumonia-like organism (PPLO)-tested by bisbenzimide staining. Reagents were from Sigma-Aldrich (Poole, UK) except where specified. Melanoma cells were maintained in RPMI 1640 medium (Invitrogen, Paisley, UK) with penicillin (10^5^ U l^−1^), streptomycin sulphate (100 mg l^−1^), glutamine (2 mM), 10% foetal calf serum (FCS) (Invitrogen) and extra Phenol Red (7.5 µg ml^−1^). Normal melanocytes and Hermes 3c immortal melanocytes were grown in the same medium but with cholera toxin (200 pM), tetradecanoyl phorbol acetate (TPA) (200 nM), human stem cell factor (10 ng ml^−1^) and endothelin 1 (10 nM). Human fibroblasts, HeLa cells and other cancer lines were grown in Dulbecco's modified Eagle’s Medium (DMEM) (Invitrogen) and 10% FCS. Subculture was with EDTA and trypsin, at appropriate concentrations for different cell types.

### Cell growth assays

Custom peptides were synthesized and provided by Bio-Synthesis Inc. (Lewisville, TX, USA). The sequences are as follows. All sequences start at amino acid 84 of human p16. Residues that differ from the normal p16 sequence are underlined.

P16P1: NH_2_-DAARE GFLAT LVVLH RAGAR RRRRR RRR-OH

P16P2: NH_2_-DAARE GFLDT LVVRH RAGAR RRRRR RRR-OH

P16P3: NH_2_-DAARE GFLAT LVVLH RAGAR-OH

Stocks were prepared at 1 mM in 1 mM acetic acid in distilled water, filter-sterilized, aliquoted and stored at −80°C. They were diluted in culture medium to obtain the concentrations stated. Cells were plated in triplicate wells of 24-well plates for each treatment in their standard culture medium. Peptides were added the same day, by a medium change after cell attachment. For vehicle controls (no peptide), acetic acid was added at 30 µM, as for 30 µM peptides. Media and additions were renewed on day 3, or day 4 for normal melanocytes. On the indicated days, cells were harvested as for subculture and three haemocytometer counts each of at least 150 cells were made per well. Cells were observed using an Olympus IMT-2 inverted microscope (Olympus UK, Southall, UK), and images captured with a 1.3-megapixel FireWire camera and PixelLINK software (Ottawa, Canada). Sample sizes in this and other assays were chosen from previous experience to show reproducible outcomes between independent experiments.

### Apoptosis assays

For TUNEL staining, triplicate cultures per treatment were harvested and the used medium from each well, containing any floating dead cells, was retained and combined with the corresponding cell suspension before making cell counts. Each suspension was centrifuged on to a glass slide in a Cytospin unit (Universal 320, Hettich Zentrifugen, GMI, MN, USA) (8 min, 110 ***g***). Cells were air-dried for several minutes, and then fixed in fresh 4% formaldehyde in PBS (10 min) followed by ethanol-acetic acid (2:1) at −20°C (5 min). They were stained using the Apoptag Plus Fluorescein *in situ* detection kit (S7111, Chemicon International, USA), according to the manufacturer's instructions. They were counterstained with bisbenzimide, mounted and viewed with an Axioplan-2 Zeiss digital imaging microscope. Positive (green) and total nuclei were counted from three representative fields (10× objective) per sample, usually by two different observers, whose counts were similar. The percentages of positive cells were calculated and averaged.

For caspase 3/7 assays, cells were plated in triplicate per treatment at 10^5^ cells per well (200 µl) of a 96-well plate (black-walled, clear-bottomed; Greiner Bio-One, Frickenhausen, Germany). After 4 h to allow attachment, half the medium was removed and replaced with fresh medium containing peptides or staurosporine solution to give the final concentrations required. After a further 24 h, the assay kit Apo-ONE Homogeneous Caspase 3/7 (G7790, Promega, Southampton, UK) was used according to the manufacturer's instructions. Fluorescence was measured in a FLUOstar Optima microplate reader (BMG Labtech, Offenburg, Germany).

## Supplementary Material

10.1242/biolopen.059965_sup1 Supplementary information
