# Supplementary material for: Preferential killing of melanoma cells by a p16-related peptide
Source: Biol Open. 2023 Aug 17;12(8):bio059965. doi: 10.1242/bio.059965 (PMC10445694; doi:10.1242/bio.059965)
Supplement: Supplementary information [file biolopen-12-059965-s1.pdf]

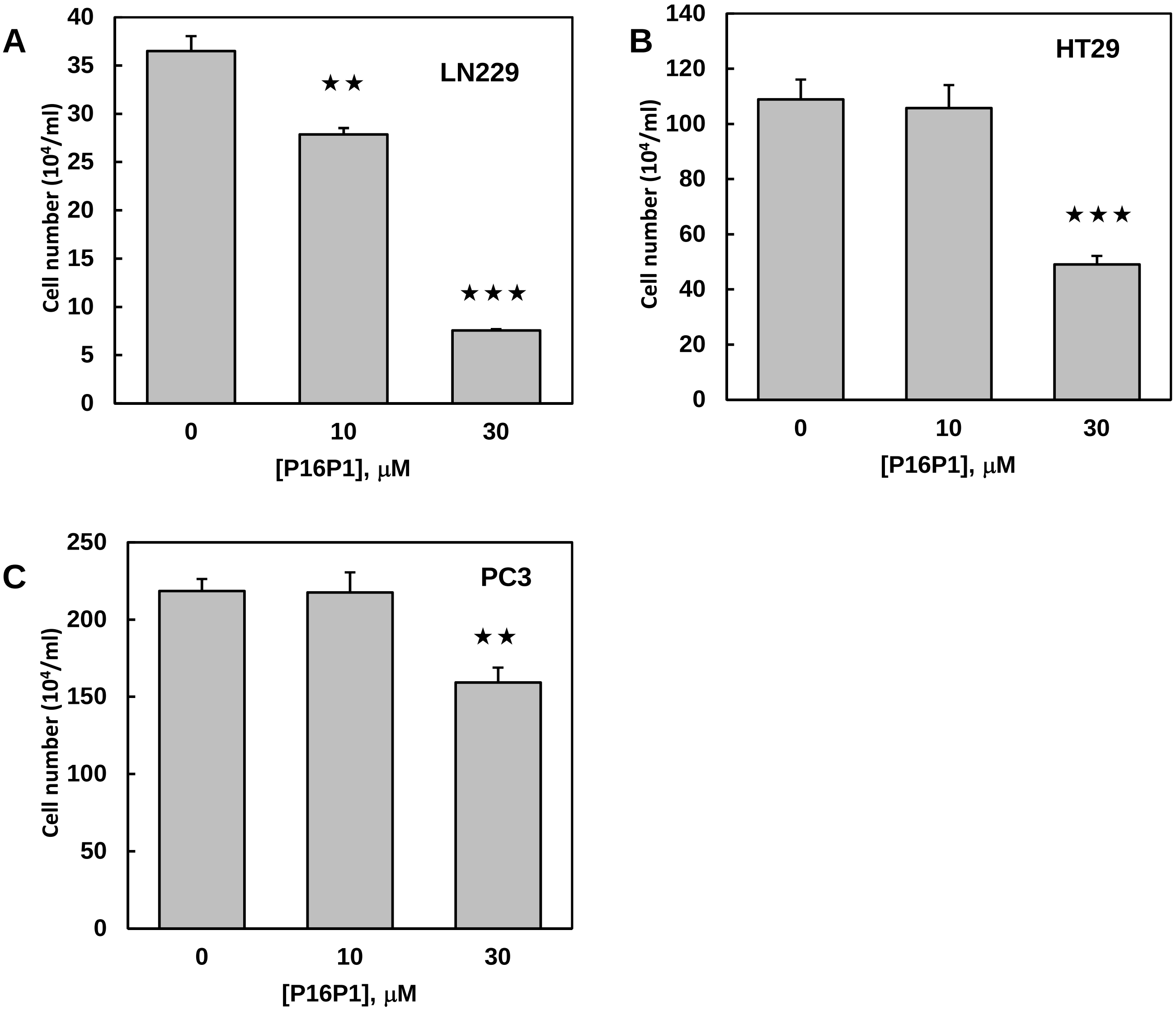

Fig. S1. Responses of other cancer cell lines to P16P1.

Cells were plated at 3 x 10^4/ml and grown with the indicated concentrations of P16P1 for 5 days with a medium change on day 3. Cells were harvested by standard methods for each line on day 5 and were counted from triplicate cultures as described (Materials and Methods). Means and SEM are shown. The cell lines tested were: (A) LN229 glioblastoma cells, (B) HT29 colon carcinoma cells and (C) PC3 prostate carcinoma cells. All were grown in DMEM with 10% FCS and 10% CO_2. These were kindly provided by respectively: Dr Soo-Hyun Kim (St George's, University of London), Professor W Nicol Keith (University of Glasgow) and Dr Ferran Valderrama (St George's, University of London). Stars indicate significant differences from 0 peptide (vehicle control). (**) p<0.01; (***) p<0.001.

Biology Open • Supplementary information

Table S1. Status of p16 pathway genes/proteins in cell lines used

**Cell line**	**p16 (*CDKN2A*)**	***CDK4***	***RB* family**
Melanoma 451Lu[1]	Defective (NFS)	WT (but cyclin D1 copy gain)	N
Melanoma WM239a[1,2]	Homozygous deletion	WT	N
Melanoma WM1158[1]	Defective (point mutations)	WT	N
Immortal melanocytes Hermes 3c[3]	Assumed WT	Assumed WT	Inactivated by HPV-16 E7
HeLa cervical carcinoma	WT, overexpressed in response to RB family dysfunction	WT, repressed by high endogenous p16	Inactivated by HPV-18 E7
PC-3 prostate carcinoma[2]	Repressed by methylation	WT	WT
HT29 colorectal adenocarcinoma[2]	Repressed by methylation, weak expression	WT	WT
LN229 glioblastoma[2,4]	Homozygous deletion	WT	WT
Normal melanocytes: Nohm1, 830c	Assumed WT	Assumed WT	Assumed WT
Normal dermal fibroblasts: Hfib	Assumed WT	Assumed WT	Assumed WT

N: no abnormality reported. NFS: not further specified. WT: wild-type (normal). All cells are human.

[1]Data from Wistar Institute website (source of lines).
https://wistar.org/sites/default/files/2017-11/Herlyn%20Lab%20-%20Cell%20Lines.xlsx
Viewed 26/06/2023

[2]Data from COSMIC database (whole exon sequencing). COSMIC | Catalogue of Somatic Mutations in Cancer (sanger.ac.uk). Viewed 10/07/2023.

[3]Line Hermes 3c was immortalized from Nohm1 melanocytes by viral transduction of TERT and HPV16-E7 (Gray-Schopfer et al, 2006, see main text).

[4]**Ishii N., Maier D., Merlo A., Tada M., Sawamura Y., Diserens A. C. and Van Meir E. G.** (1999). Frequent co-alterations of TP53, p16/CDKN2A, p14ARF, PTEN tumor suppressor genes in human glioma cell lines. *Brain Pathol.* **9,** 469-479.
